# Temporal dynamics of the cecal and litter microbiome of chickens raised in two separate broiler houses

**DOI:** 10.3389/fphys.2023.1083192

**Published:** 2023-03-02

**Authors:** Benjamin Zwirzitz, Adelumola Oladeinde, Jasmine Johnson, Gregory Zock, Marie C. Milfort, Alberta Lorraine Fuller, Ahmed F. A. Ghareeb, James C. Foutz, Jose Alexis Teran, Reed Woyda, Zaid Abdo, Torey Looft, Jodie Plumblee Lawrence, Denice Cudnik, Samuel E. Aggrey

**Affiliations:** ^1^ Department of Food Science and Technology, Institute of Food Science, University of Natural Resources and Life Sciences, Vienna, Austria; ^2^ Austrian Competence Centre for Feed and Food Quality, Safety and Innovation FFoQSI GmbH, Tulln, Austria; ^3^ USDA-ARS, U.S. National Poultry Research Center, Athens, GA, United States; ^4^ Department of Poultry Science, University of Georgia, Athens, GA, United States; ^5^ College of Engineering, University of Georgia, Athens, GA, United States; ^6^ Department of Microbiology, Immunology and Pathology, Colorado State University, Fort Collins, CO, United States; ^7^ Program of Cell and Molecular Biology, Colorado State University, Fort Collins, CO, United States; ^8^ USDA-ARS, National Animal Disease Center, Ames, IA, United States

**Keywords:** broiler chickens, microbiome, antimicrobial resistance (AMR), pre-harvest, environmental condition

## Abstract

In this study, we investigated the dynamics of the ceca and litter microbiome of chickens from post-hatch through pre-harvest. To achieve this, six hundred one-day old Cobb 500 broiler chicks were raised on floor pens for 49 days in two separate houses. We performed short-read and full-length sequencing of the bacterial 16S rRNA gene present in the meconium and in cecal and litter samples collected over the duration of the study. In addition, we determined the antimicrobial resistance (AMR) phenotype of *Escherichia coli* and *Enterococcus* spp. isolated from the meconium and the ceca of 49-day old chickens. We monitored the relative humidity, temperature, and ammonia in each house daily and the pH and moisture of litter samples weekly. The overall microbial community structure of the ceca and litter consistently changed throughout the course of the grow-out and correlated with some of the environmental parameters measured (*p* < 0.05). We found that the ceca and litter microbiome were similar in the two houses at the beginning of the experiment, but over time, the microbial community separated and differed between the houses. When we compared the environmental parameters in the two houses, we found no significant differences in the first half of the growth cycle (day 0–21), but morning temperature, morning humidity, and ammonia significantly differed (*p* < 0.05) between the two houses from day 22–49. Lastly, the prevalence of AMR in cecal *E. coli* isolates differed from meconium isolates (*p* < 0.001), while the AMR phenotype of cecal *Enterococcus* isolates differed between houses (*p* < 0.05).

## Introduction

Broiler house environment is one of the most important management factors that has been shown to significantly affect broiler performance, welfare, and health (Winn and Godfrey, 1967; Deaton et al., 1978; Weaver and Meijerhof, 1991; Jones et al., 2005; Bessei, 2006; Wei et al., 2015; Baracho et al., 2018; Nassem and King, 2018). Temperature and relative humidity of a broiler house are interconnected factors that affect litter moisture and emitted ammonia (Ritz et al., 2005). Together, these environmental parameters have been shown to influence broiler growth, feed conversion efficiency, disease etiology, occurrence of pathogens and in some cases, mortality (Miles et al., 2004; Ritz et al., 2004; Bessei, 2006; De Jong et al., 2014; Wei et al., 2015; Baracho et al., 2018). The results from these earlier studies served as the framework for broiler management/husbandry guidelines used by the poultry industry (Donald, 2010; Vantress, 2013). Therefore, there is sufficient data supporting the importance of proper environmental management.

Contrastingly, there is limited data on how changes in environmental factors affect the microbiome of broiler chickens. The few studies that have investigated the role of the environment have focused on exposing broilers to an environmental stressor e.g., temperature or ammonia (Wang et al., 2018; Zhou et al., 2021c; Han et al., 2021; Yang et al., 2021; Emami et al., 2022). Broiler chickens exposed to temperature levels that induce heat stress harbored a different bacterial community structure in the ceca compared to non-stressed control chickens (Shi et al., 2019; Liu et al., 2022). Similarly, exposing broilers to 25–35 ppm of ammonia was reported to alter the microbiota of the trachea (Zhou et al., 2021b). Changes in litter moisture and pH have been shown to perturb the microbiome of litter and affect the survival of bacterial pathogens including *Salmonella* (Lovanh et al., 2007; Payne et al., 2007; Chinivasagam et al., 2012; Dunlop et al., 2016; Bucher et al., 2020). Kers et al. (2019) showed that the microbial diversity in the ceca of broilers was influenced by the type of house and resulted in significant variability in the interventions tested.

Other studies have focused on the litter and its interaction with the gastro-intestinal tract (GIT) microbiome of broiler chickens and the occurrence of pathogens (Cressman et al., 2010; Roll et al., 2011; Roberts et al., 2013; Wang et al., 2016). For instance, broilers raised on fresh litter were shown to harbor a different microbiome compared to chicks raised on reused litter (Cressman et al., 2010; Wang et al., 2016; Oladeinde et al., 2022). Our research group (Oladeinde et al., 2022) and others (Fanelli et al., 1970; Corrier et al., 1992) have also reported that chickens grown on reused litter are less likely to be colonized by *Salmonella* than chickens on fresh litter. Taken together, these studies support the hypothesis that changes in environmental conditions during grow-out will affect the microbiome in the GIT and litter of chickens raised.

Therefore, our objectives for this study were 3-fold: i) determine the temporal changes in the GIT and litter microbiome of broiler chickens from post-hatch to pre-harvest ii) determine environmental parameters that correlated with changes in the microbiome of broiler chickens and iii) evaluate if changes in the microbiome and environment resulted in bacterial strain-level changes in antimicrobial resistance (AMR) phenotype. Our results revealed that the overall microbial community structure of the ceca and litter consistently changed throughout the course of the grow-out and that these changes correlated with some of the environmental parameters measured in the two different houses. We found no significant differences in environmental parameters between the houses in the first half of the grow-out (day 0–21), but morning temperature, morning humidity, and ammonia significantly differed between houses from day 22–49. The AMR phenotype of cecal *Escherichia coli* isolates differed from the meconium isolates, while the AMR phenotype of cecal *Enterococcus* isolates differed between the houses.

## Materials and methods

### Study design

**FIGURE 1:**
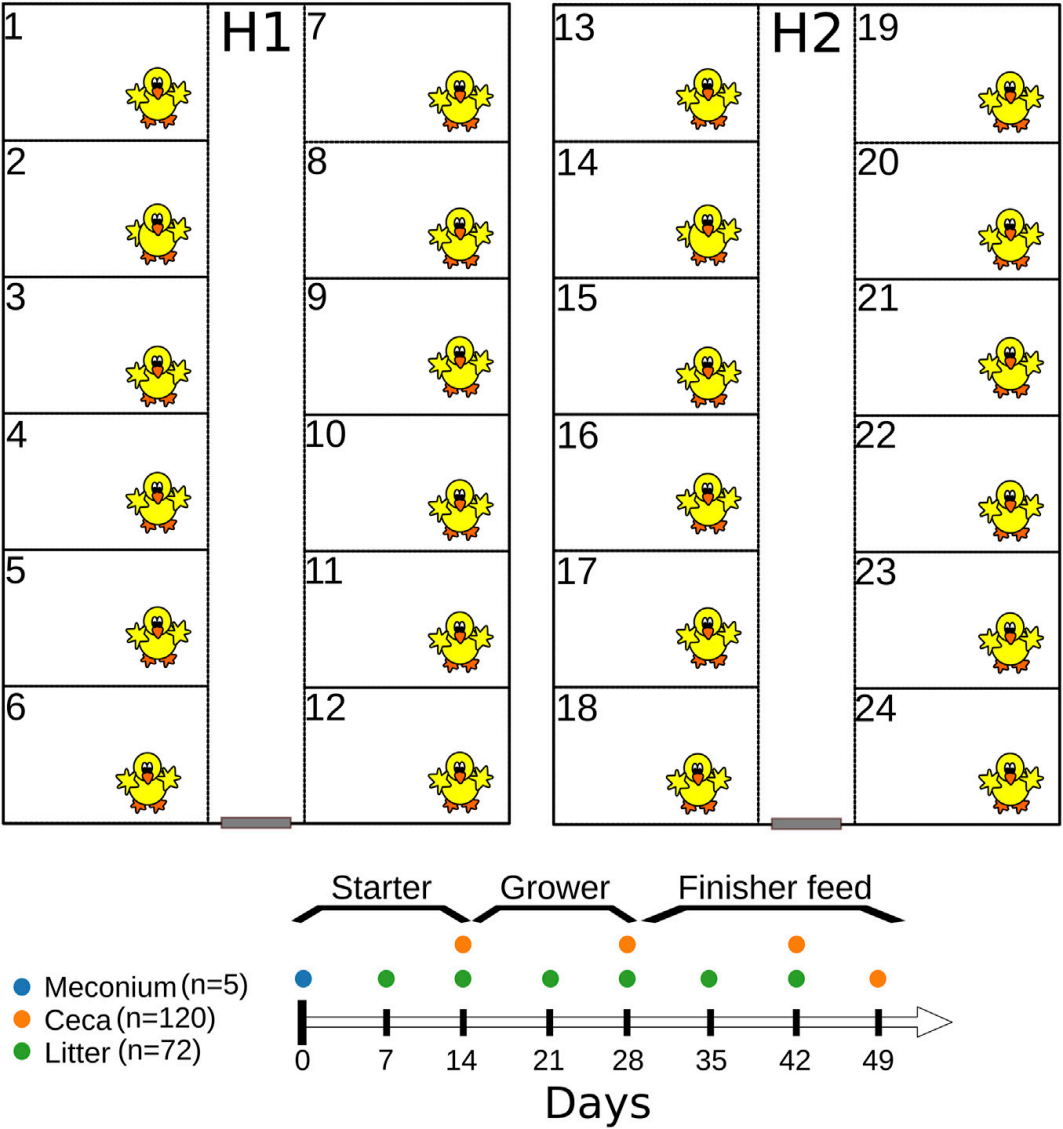
Graphical representation of the experimental design.

Six hundred 1-day old Cobb 500 broiler chicks were raised in two separate houses (H1 and H2) for 49 days (Figure 1) both located at the experimental farm of the University of Georgia (33.907101 N 83.380368 W). Before chick placement, each house was cleaned-out and steamed. Broiler chicks were raised in floor pens (12 pens/house, 25 chicks/pen) measuring 1.84 m (length) L by 1.16 m width, and fresh pine shavings were used as the bedding material (Figure 1). Broiler chickens were given water and feed *ad libitum* and were raised antibiotic-free on starter (days 0–15), grower (days 15–29), and finisher (days 29–49) feeds (feed was synthesized by the University of Georgia’s Poultry Research Center’s feed mill). On day 49, feeders were removed from 6 pens in each house for 8 h before all chickens were euthanized. Husbandry and management followed commercial broiler chicken industry guidelines. Chicken mortality was recorded daily while body weights were measured on day 0, 14, 28, 42 and 49. Additionally, we used Portacool evaporative fans (Port-A-Cool, L.L.C., Center, TX; model PAC2K24HPVS) to reduce the air temperature when the house temperature was above 85°F. Broiler chickens were euthanized as approved by the University of Georgia Office of Animal Care and Use under Animal Use Protocol (A2018 05–013-R1) before cecal sampling and at the completion of the study. The study was conducted from 11 July 2019–29 August 2019.

### Determination of environmental parameters

Litter moisture was determined gravimetrically while litter pH was determined using a Thermo-Scientific Orion probe (ThermoFisher Scientific) as described before (Johnson et al., 2021). Broiler house ammonia levels were monitored by attaching ammonia dosimeter tubes (Gastec Corporation) to metal chains hung at about 37 cm ± 5.4 cm from the litter floor of three pens from each house (Johnson et al., 2021). Readings on ammonia tubes were recorded ∼7.5 h after installation and performed once a week. The pen used for monitoring ammonia changed weekly for each house. The temperature and relative humidity reading inside each house was recorded from thermostats (Temperature, Johnson Controls, Inc., Milwaukee, WI, model: A419 Temperature Control with NEMA 4X, Enclosure and A99 Temperature Sensor; Humidity, AcuRite Lake Geneva, WI, model: AcuRite^®^ indoor digital thermometer and hygrometer 00609SBLA) installed in each house at the same time in the mornings (7 a.m.–10:00 a.m.) and afternoons/evenings (5 p.m.–9 p.m.). Litter moisture, pH and ammonia levels were measured weekly, while temperature and humidity were measured daily, resulting in different sample sizes for these indicators.

### Meconium, cecal and litter sampling

Chick pads used for transporting 1-day old broiler chicks from the hatchery were used for meconium (the dark greenish-yellow first droppings of a chick) recovery. In addition, chick pads (*n* = 5) that were not used for chick transportation were included as controls. Each chick pad (*n* = 5) was transferred into a 1-gallon Whirl Pak bag and 500 mL of buffered peptone water (BPW) was added. Afterwards, the bag was shaken by hand for 2 min and incubated for 1 h at 37°C. After incubation, 200 mL aliquots of the mixture were transferred to 250 mL Nalgene bottles and centrifuged at 4,600 g for 10 min. Thereafter the supernatant was decanted, and the pellet was resuspended in an equal volume of Luria Bertani (LB) broth containing 60% glycerol (final glycerol concentration was 30%), vortexed and saved in cryovials at −80°C. Pellets saved in LB glycerol were used for 16S rRNA gene sequencing and the retrospective isolation of *E. coli* and *Enterococcus* spp.

Cecal contents were collected from chickens (*n* = 120) on days 14, 28, 42, and 49 (Figure 1). Briefly, floor pens were given numbers at the start of the experiment (1–24), and six odd- or even-numbered pens/house were randomly sampled on day 14 and 28. For example, on day 14, we selected one chicken each from six odd-numbered pens from each house (*n* = 12 per sampling day), while on day 28, chickens were selected from six even-numbered pens from each house (*n* = 12). On day 42 and 49, two chickens were selected from each pen from each house (*n* = 48 for each sampling day). The weight of individual chickens was measured before the ceca were removed from the eviscera. Thereafter, the ceca were stomached for 60 s after the addition of 3 × volume to the weight (vol/wt) of BPW. Cecal contents were resuspended in an equal volume of LB broth containing 60% glycerol, vortexed and saved in cryovials at −80°C. Cecal contents saved in LB glycerol were used for 16S rRNA gene sequencing and the retrospective isolation of *E. coli* and *Enterococcus* spp. from day 49 samples.

Litter samples were collected from floor pens on days 7, 14, 21, 28, 35 and 42 (Figure 1). Litter was collected as grab samples from seven locations in each pen, including the corners and under the waterers (Johnson et al., 2021). On each sampling day, litter was collected from six odd- or even-numbered pens per house (Johnson et al., 2021) (*n* = 72). Litter samples were mixed thoroughly by hand in the Whirl Pak bags and processed as described previously (Johnson et al., 2021). Briefly, 10 g of litter were placed in a Nalgene bottle containing acid-washed glass beads covering the bottom of bottle (S800242, ThermoFisher Scientific), and 50 mL of 1 × phosphate-buffered saline (ThermoFisher Scientific) was added. Sample bottles were mixed on an automatic wrist shaker (Boekel Scientific) at 450 rpm for 10 min and allowed to rest upright for 5 min after shaking (Johnson et al., 2021). Five milliliter of the eluate was transferred into LB broth containing 60% glycerol, vortexed and saved in cryovials at −80°C. Eluate saved in LB glycerol was used for 16S rRNA gene sequencing.

### DNA extraction and sequencing

DNA was extracted from 250 μL of LB glycerol containing either meconium, cecal contents or litter eluate using the Qiagen DNeasy PowerLyzer Powersoil kit (Qiagen Inc., MD, United States) according to manufacturer instructions. In addition, DNA was extracted from 13 negative controls (5 Chick pad paper and 8 DEPC-treated H_2_O samples). Amplicon sequencing libraries for all samples were generated as previously described (Allen et al., 2016). Briefly, the V4 hypervariable region of the bacterial 16S rRNA gene was PCR amplified and sequenced using the paired-end (250 × 2) method on the Illumina MiSeq platform. Additionally, 62 samples (24 cecal, 24 litter, 5 meconium, 5 chick pad paper, and 4 negative control samples) were sequenced on the Pacbio Sequel II platform to get the full-length of the 16S rRNA gene for better species classification. Preparation and sequencing of full-length (V1–V9) 16S rRNA gene libraries were done by the sequencing core center of University of Georgia (Athens, GA, United States) as described previously (Schloss et al., 2016). Raw sequence reads are available under NCBI accession number PRJNA699167.

### 16S rRNA sequence processing and data analysis

Raw sequence reads obtained from the Illumina Miseq were processed in R using the DADA2 package (version 1.14) (Callahan et al., 2016). Only reads with a maximum number of expected errors lower than or equal to 2 were retained. In addition, reads were truncated where the phred quality score dropped below 30. Chimeras were identified and removed using the consensus method and the remaining reads were annotated to the SILVA database release 138 with a minimum bootstrap threshold of 50 (Quast et al., 2013). Additionally, full-length 16S rRNA gene sequences generated on the Pacbio Sequel II were processed in the SMRT Link software package version 8.0. The circular consensus reads (ccs) were determined with a minimum predicted accuracy of 0.99 and the minimum number of passes set to 3. After demultiplexing, the ccs were further processed with DADA2 (version 1.14) to obtain high quality amplicons with single-nucleotide resolution as previously described (Callahan et al., 2019). Same as the Illumina reads, the full-length 16S rRNA gene sequences were annotated to the SILVA database 138. Hereafter, the annotated Pacbio reads were used to create a custom formatted database that was utilized as a reference for the Illumina reads that were generated from the same samples. Iterating the species taxonomy assignment of the Illumina reads to the custom database and adding this information to the taxonomy table improved species classification rate by 35%. Amplicon sequence variants (ASVs) with less than 5 sequences in total were removed from the dataset before decontamination. Contaminant sequences were identified from extracted negative controls with the R package decontam and the probability threshold set to 0.5. After contaminant removal, samples with less than 1,000 sequences were removed. The average sequence depth per sample was 23,088.38, ranging from 1,769 to 93,023 sequences.

In-depth microbial community analysis was performed in the R environment using the packages “phyloseq”, “Ampvis2”, “vegan”, and “MaAsLin2”. Alpha diversity indices were calculated with a dataset rarefied to the smallest sample size. Values of alpha diversity indices were checked for normal distribution by visually assessing qqplots and histograms and by calculating the Shapiro-Wilk normality test. The groups that were not normally distributed were compared using the Wilcoxon Signed Rank test. A non-metric multidimensional scaling ordination based on Bray-Curtis distances was performed to calculate changes in microbial beta diversity. In addition, a permutational multivariate analysis of variance (PERMANOVA) was performed to assess the influence of experimental factors on the microbial community in ceca and litter samples. Prior to this analysis, ASV’s that are not present in more than 0.1% relative abundance in any sample have been removed. ASVs were considered part of the core microbiome with a relative abundance cutoff above 0.01% and a prevalence cutoff above 80% of the samples. Temporal microbial shifts and differences of ASVs between houses were computed using MaAsLin2. Only associations for ASVs with a minimum prevalence of 10% and a minimum relative abundance of 1% were calculated. For temporal microbial shifts the variables “Pen” and “House” were set as random effects, while for differences between houses only “Pen” was set as a random effect. Benjamini–Hochberg procedure was applied as a correction method for computing the q-values.

### Retrospective isolation of *E. coli* and *Enterococcus* spp. from meconium and cecal samples

One hundred microliters of meconium samples (*n* = 5) and 10 µL of cecal samples (*n* = 48) previously saved in LB glycerol at −80°C were vortexed and spread plated onto CHROMagar™ ECC (DRG International, Inc., Springfield, NJ). CHROMagar™ ECC was incubated for 18 h–24 h at 37°C and 5 isolated blue-green colonies typical of *E. coli* were subcultured for isolation to a fresh CHROMagar™ ECC and incubated as above. For *Enterococcus* spp. isolation, 100 µL of meconium and cecal samples was spread plated onto mEnterococcus agars (Neogen, Lansing, MI). mEnterococcus agar was incubated for 48 h at 37°C and 5 pink to dark red colonies indicative of *Enterococcus* spp. were re-struck for isolation to a fresh mEnterococcus agar and incubated as indicated.

After isolation of *E. coli* and *Enterococcus* spp. on selective agar, all isolated colonies were subcultured to Tryptic Soy Agar with 5% sheep blood (BAP) agar (Remel, Lenexa, KS), incubated 18 h at 37°C and then re-struck to BAP. Isolate identification was confirmed using qPCR on a CFX96 Touch Real-Time System (Bio-Rad, Hercules CA). Primers (Ludwig and Schleifer, 2000; Jackson et al., 2004; Chern et al., 2011) were synthesized by Integrated DNA Technologies (Coralville, IA) and are listed in Supplementary Table S1. Reaction mixtures (20 µL) for all assays contained 1X SsoAdvanced Universal SYBR Green Supermix (Bio-Rad), 600 nM (each) primers, and 4 µL of isolate whole cell template (1 colony in 100 µL nuclease-free water; boiled for 10 min). Thermal conditions for all assays except individual *Enterococcus* spp. were initial denaturation at 98°C for 3 min followed by 40 cycles of denaturation at 95°C for 15 s and an annealing/extending step at 60°C for 30 s before melting from 65°C to 95°C at 0.5°C increments. The *Enterococcus faecalis*, *Enterococcus faecium*, and *Enterococcus hirae* species assays were adapted from Jackson et al. (2004) using the cycling conditions above but decreasing the annealing temperature to 55°C. Melt curves were visually inspected to ensure standards and samples had peaks at the same temperature and no secondary peaks were formed.

### Antimicrobial susceptibility testing

Minimum inhibitory concentrations for isolates were determined by broth microdilution using the Sensititre™ semiautomated antimicrobial susceptibility system (Thermo Fisher Scientific, Waltham, MA). Using the National Antimicrobial Resistance Monitoring System (NARMS) protocol (US FDA, 2019) *E. coli* isolated from meconium (*n* = 25) and ceca (*n* = 96) were tested using the CMV4AGNF panel while *Enterococcus* spp. isolated from meconium (*n* = 25) and ceca (*n* = 90) were tested using the CMV3AGPF panel. Results were interpreted according to Clinical and Laboratory Standards Institute (CLSI) guidelines when available (CLSI, 2019); otherwise, breakpoints established by NARMS were used (US FDA, 2019).

Heatmaps were generated using the pheatmap v1.0.12 package in R. A distance matrix was generated using the jaccard metric *via* the vegdist function from the Vegan v2.6 package. Optimal number of clusters was identified using the silhouette method implemented by the fviz_nbclust function from the factoextra v1.0.7 package. Hclust () from the stats v3.6.2 package was then utilized to perform hierarchical clustering under the “complete” method using the determined optimal number of clusters. All analyses were done in R v4.0.4 utilizing RStudio v1.2.1106.

### Statistical analyses

The measured environmental parameters were tested for normal distribution by calculation of the Shapiro–Wilk normality test and visually as histograms and Q-Q plots. Normal distributed parameters were compared using a student *t*-tests and not normal distributed parameters were compared using a pairwise Wilcoxon signed rank test. *p* values were corrected with the Benjamini–Hochberg method. Wilcoxon rank sum test was performed to determine if there were significant differences between sample type (meconium vs. ceca) and houses (house 1 vs. 2) in the number of antibiotic drug classes and antibiotic drugs *E. coli* and *Enterococcus* isolates were resistant to. *p* values were corrected with the Benjamini–Hochberg method. Statistical comparisons were performed using R v4.2.0 using the stats v3.6.2 package.

## Results

### Microbial diversity of litter and ceca increased throughout grow-out

**FIGURE 2:**
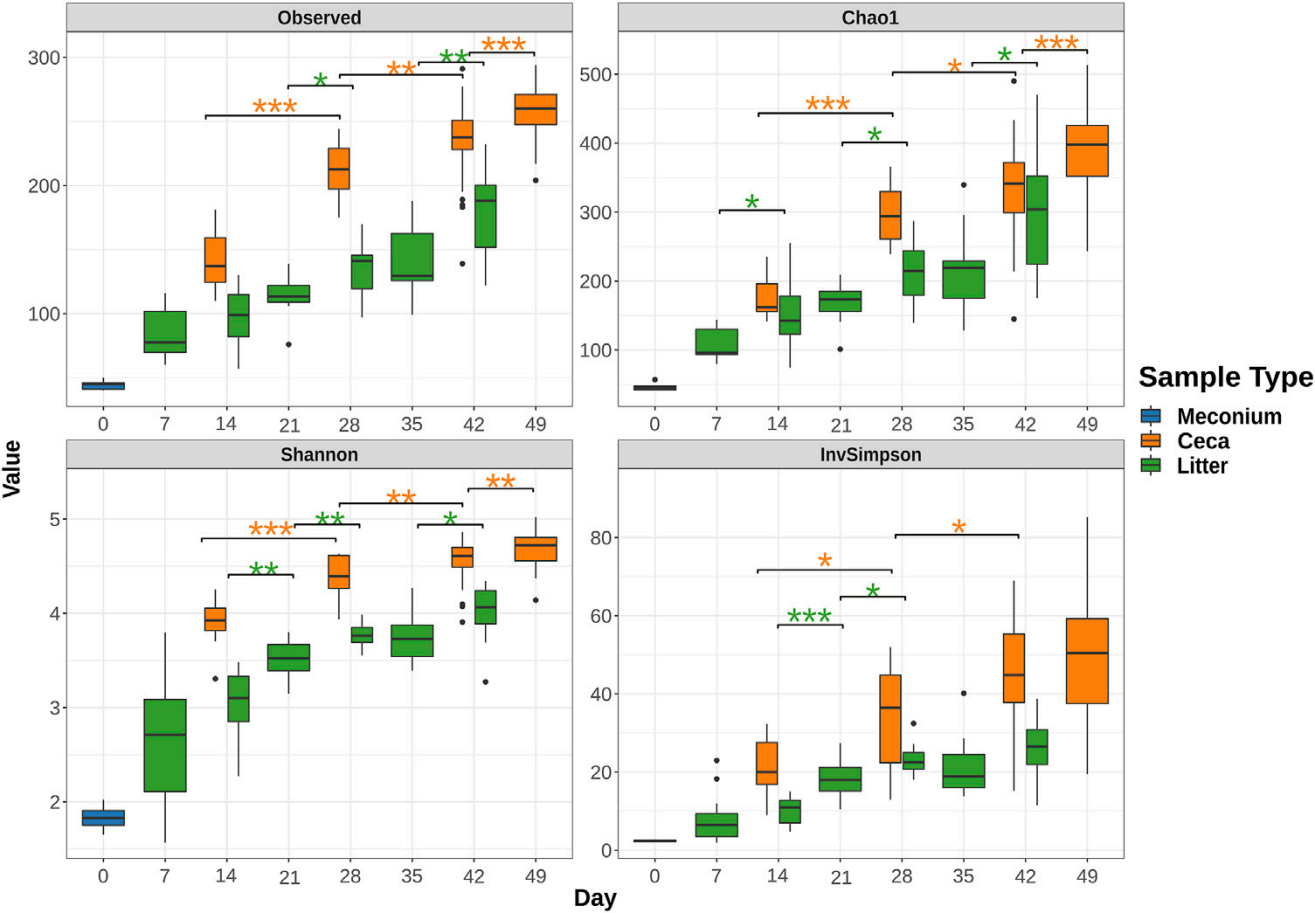
Change of alpha diversity indices from rarefied 16S rRNA gene libraries over time. Boxes indicate the interquartile range (75th to 25th) of the data. The median value is shown as a line within the box. Whiskers extend to the most extreme value within 1.5 * interquartile range and dots represent outliers. Only significant changes are shown with asterisks: *: *p* ≤ 0.05, **: *p* ≤ 0.01, ***: *p* ≤ 0.001. The color of asterisks shows pairwise comparisons between ceca (orange) or litter (green) samples.

Meconium samples showed the lowest species richness of all sample types with an average of 44.4 amplicon sequence variants (ASVs). In comparison, ceca and litter samples harbored an average of 85.0 ASVs and 142.3, respectively on day 7 and 14. The number of observed ASVs increased significantly over the course of the study, reaching 258.6 ASVs in cecal (day 49) and 180.6 ASVs in litter (day 42) samples (Figure 2). The Shannon and Inverse Simpson indices of alpha diversity showed that the diversity of the ceca and litter microbiome increased from the start to end of the grow-out. Furthermore, chickens raised in house 2 had higher cecal alpha diversity (Observed, *p* = 0.026; Shannon, *p* = 0.041; Simpson, *p* = 0.13) than chickens in house 1 at day 28, while chickens in house 1 had higher alpha diversity (Observed, *p* < 0.001; Shannon, *p* < 0.001; Simpson, *p* < 0.001) at day 49 (Supplementary Figure S1). Similarly, litter from house 2 had higher alpha diversity than house 1 at day 28, while litter from house 1 had higher alpha diversity than house 2 at day 49, however, these differences were not statistically significant (Supplementary Figure S1).

### Environmental factors, chicken weight, mortality and the bacterial community of the ceca and litter differed between houses

We found that chicken body weights differed between houses at the end of the grow-out (Supplementary Figure S2A). The average weight of 1-day old chicks was 43.04 ± 0.73 g and there was no significant difference (*p* > 0.05) in weight between chicks placed in house 1 compared to house 2. However, at the end of the grow-out, chickens in house 1 (average = 3,375.58 ± 482 g) weighed more than chickens in house 2 (average = 3,035.42 ± 349 g) (*p* < 0.01). Furthermore, chickens in house 2 experienced higher premature mortality (∼6%) than chickens in house 1 (∼3%) (Supplementary Figure S2B).

**FIGURE 3:**
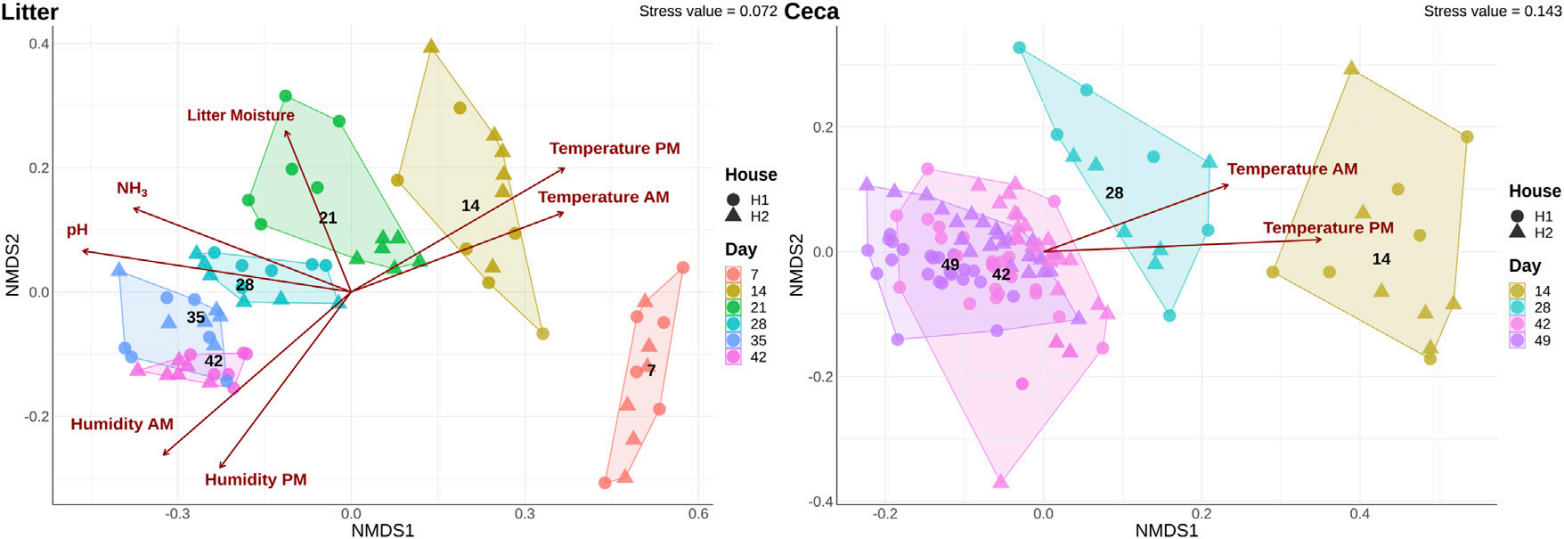
Shifts of microbial community composition in cecal and litter samples. Beta diversity was calculated using a non-metric multidimensional scaling (NMDS) ordination of 16S rRNA gene libraries based on Bray-Curtis distances. Colors show samples obtained on different days and the shape displays samples from different houses. Only significant (*p* < 0.05) environmental variables were fitted onto the ordination.

The overall bacterial community structure changed throughout the course of the grow-out in both cecal and litter samples and the changes correlated with several environmental parameters (Figure 3). Litter moisture, litter pH, house temperature, house humidity, and house NH_3_ levels were factors that explained bacterial community heterogeneity in litter samples, while in cecal samples, only house temperature was found to correlate with changes in community structure. Additional factors that explained the variation in bacterial community composition were sample type, day, and house (Table 1). Since the house was determined as a significant factor affecting bacterial community heterogeneity, we calculated analysis of similarities (ANOSIM) tests for each individual day and sample type.

**TABLE 1:** Results from a PERMANOVA test for the influence of sampling groups.

	Df	SumsOfSqs	MeanSqs	F.Model	R2	Pr (>F)
Sample type	1	15.974	15.974	83.601	0.247	<0.001***
Day/age	6	12.844	2.141	11.203	0.199	<0.001***
House	1	0.924	0.924	4.835	0.014	<0.001***
Pen	22	4.344	0.197	1.033	0.067	0.344,931
Residuals	160	30.572	0.191		0.473	
Total	190	64.657			1	

Df = Degrees of Freedom, SumsOfSqs = Sum of Squares, MeanSqs = Mean Squares, F. Model = Pseudo-F, R2 = coefficient of determination, Pr (>F) = *p*-value.

The ceca and litter microbiome were similar in the two houses at the beginning of the experiment, but over time the bacterial community separated and differed between the houses (Table 2). Therefore, we compared the environmental parameters that were monitored in the two houses (Supplementary Figure S3). No significant differences were observed in the first half of the grow-out (day 0–21), but morning house temperature and humidity, and NH_3_ levels varied between the two houses throughout the second half of the grow-out (day 22–49) (Supplementary Table S2). Morning temperature (*p* < 0.01) and NH_3_ levels (*p* < 0.05) were significantly higher in house 2 (H2), while morning humidity (*p* < 0.05) was higher in house 1 (H1).

**TABLE 2:** Results from an analysis of similarities (ANOSIM) test between the two different houses.

	Day/age	R-value	*p*-value
Litter	7	0.0130	0.3779
	14	0.3278	0.0022
	21	0.7037	0.0020
	28	0.3463	0.0154
	35	0.5907	0.0026
	42	0.6981	0.0032
Ceca	14	0.0167	0.3747
	28	0.2815	0.0224
	42	0.2877	0.0002
	49	0.3216	0.0002

**FIGURE 4:**
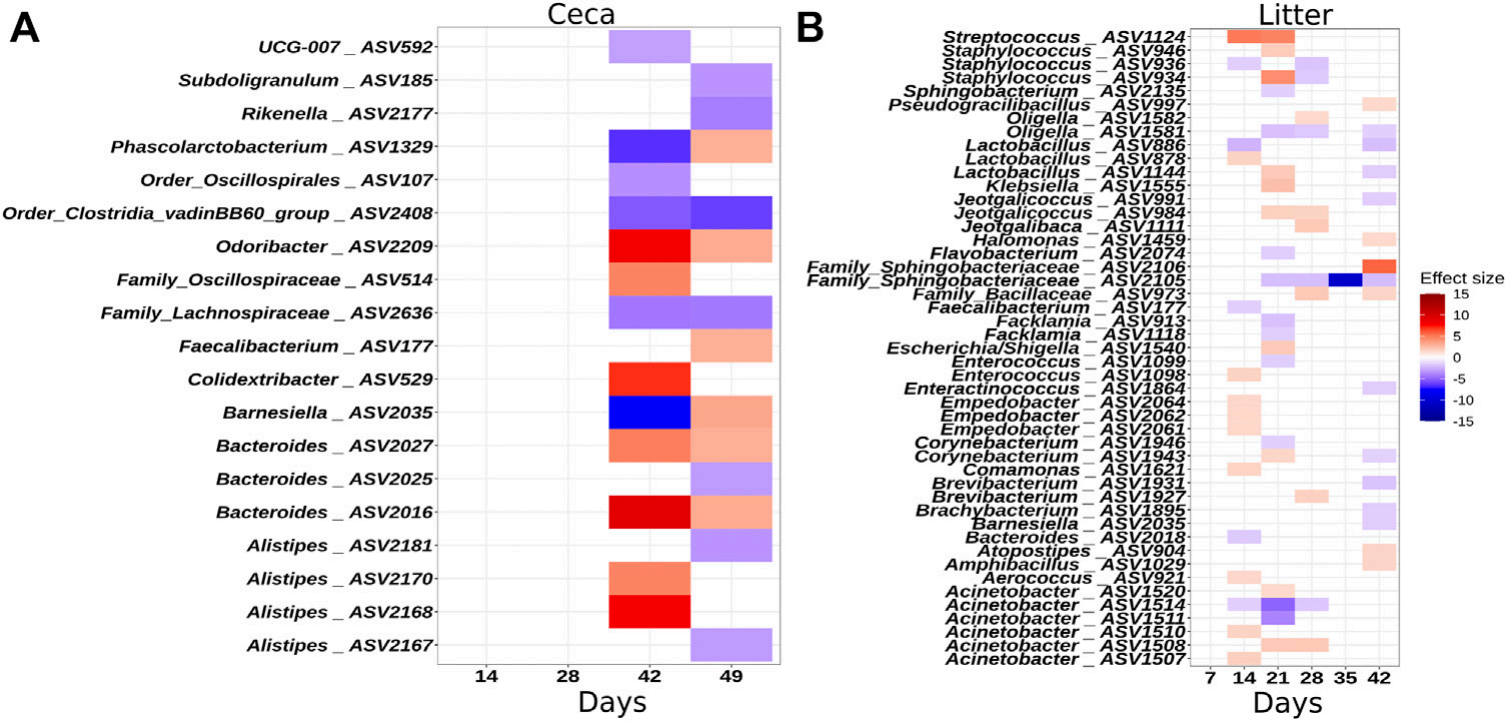
Heatmaps illustrating ASVs that were significantly differentially abundant between houses. **(A)** Ceca samples. **(B)** Litter samples. The effect size depicts the negative log of the q-value multiplied by the sign of the coefficient. A positive effect size denotes higher abundance in house 2. A negative effect size denotes higher abundance in house 1. Taxonomy of the ASVs is indicated at the genus levels or at the lowest rank that could be assigned confidently (Bootstrap support above 50). Only significant changes are shown.

To identify individual ASVs that contributed to the observed difference in microbial community composition between the houses, we performed a multivariable association analysis using MaAsLin2. In congruence with the results of the ANOSIM test, no ASVs were different in relative abundance on day 7 in litter samples, but from day 14 onwards, several ASVs were significantly more or less abundant in H1 compared to H2 (Figure 4A). Similarly, no ASVs were different at the beginning of grow-out in ceca samples, but several ASVs were different on day 42 and 49 (Figure 4B).

#### Microbial community profiles of the meconium, ceca, and litter

**FIGURE 5:**
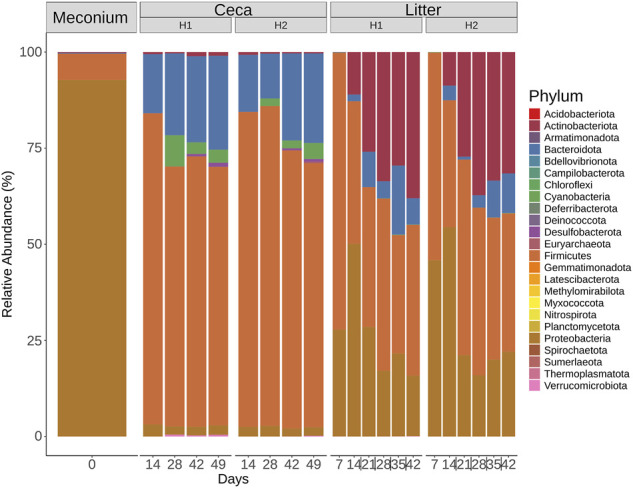
Phylum-level classification of 16S rRNA gene sequence reads. Samples were grouped by days and split by sample type (Meconium, Ceca, Litter). Relative abundance was normalized by total sum scaling and the mean for each sample group is depicted.

Six different phyla were detected in the meconium, 12 phyla in the ceca, and 15 phyla in litter samples (Figure 5). The community profiles differed between the 3 sample types. For example, meconium samples were dominated by Proteobacteria, Firmicutes and the less abundant phyla Actinobacteriota, Bacteroidota, Deinococcota, and Verrucomicrobiota. Cecal samples were composed of Firmicutes and Bacteroidota and to a lesser extent of Cyanobacteria, Proteobacteria, and other phyla with a mean relative abundance of less than 1%. The composition of the microbial communities in litter samples was different. Here, Firmicutes, Proteobacteria, Actinobacteria, and Bacteroidota were the dominant phyla throughout the grow-out. Interestingly, Actinobacteriota increased from 0.4% relative abundance on day 7%–34.81% relative abundance on day 42 in litter samples. Similarly, Bacteroidota were found at low levels at the beginning and at higher levels at the end of the grow-out.

**FIGURE 6:**
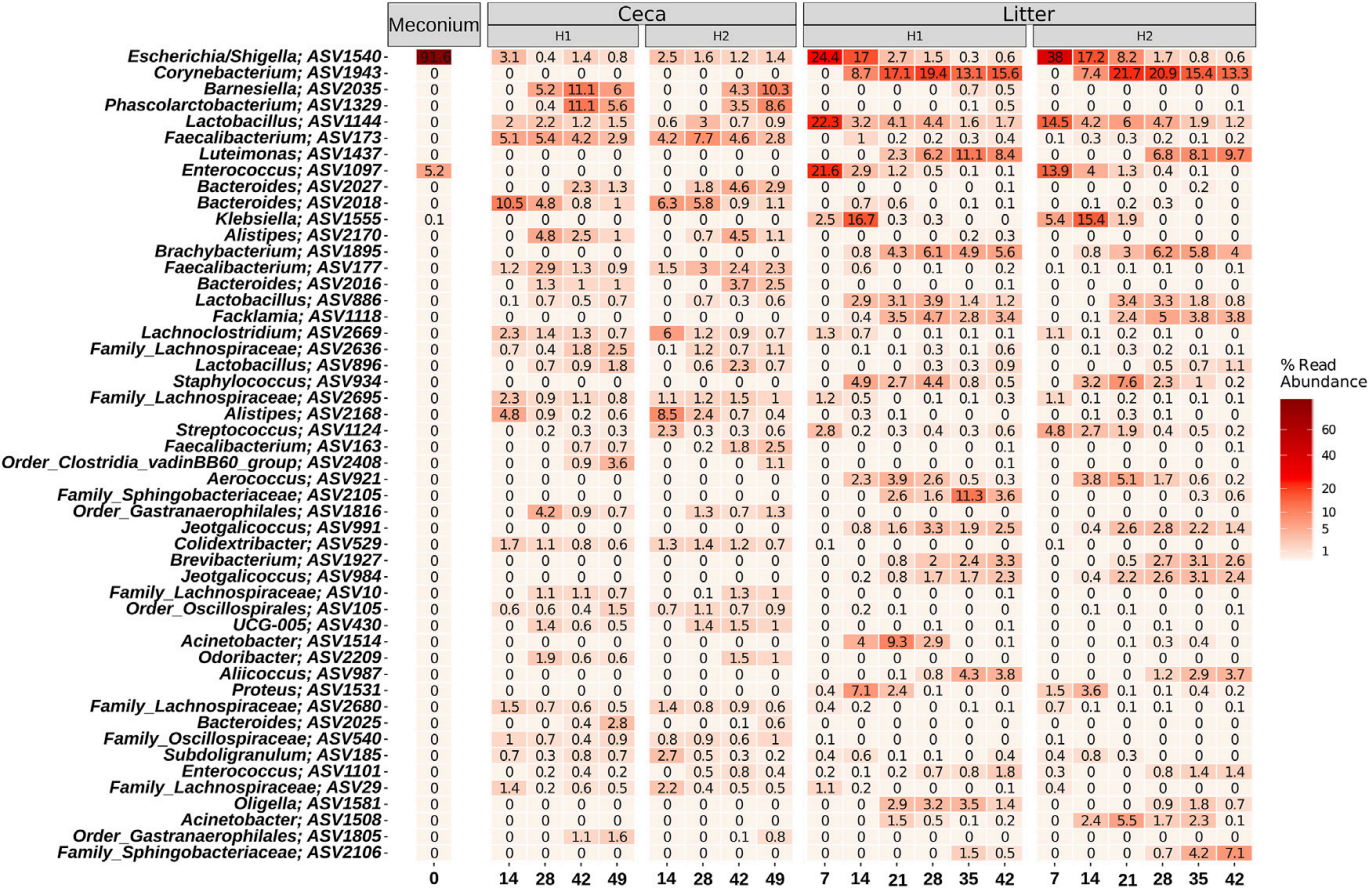
Heatmap showing the relative abundance of the 50 most abundant ASVs. Samples were grouped by days and split by sample type (Meconium, Ceca, Litter). Relative abundance was normalized by total sum scaling and the mean for each sample group is depicted. Taxonomy of the ASVs is indicated at the genus levels or at the lowest rank that could be assigned confidently (Bootstrap support above 50).

On a finer taxonomic resolution, meconium samples were dominated by two ASVs classified as *Escherichia/Shigella* and *Enterococcus* (Figure 6). Both ASVs were also highly abundant in litter samples at the beginning of the grow-out, but their abundance decreased over time. The most abundant bacteria were different between cecal and litter samples. For example, *Barnesiella*, *Phascolarctobacterium*, *Faecalibacterium*, *Bacteroides*, and *Alistipes* were found in high numbers in cecal samples, but not in litter samples. Highly abundant ASVs in litter samples were classified as *Corynebacterium, Lactobacillus, Luteimonas*, and *Klebsiella*.

Eighty ASVs were part of the core microbiome in both houses (Supplementary Figure S4). The core ASVs in cecal samples were classified as Bacilli, Bacteroidia, and Clostridia while in litter samples they were identified as Bacilli, Clostridia and Actinobacteria (Supplementary Table S3). Furthermore, we found 59 ASVs to differ between house 1 and house 2. For example, 23 ASVs were exclusively part of the core microbiome of the cecal samples of house 1, but not of house 2 and 21 ASVs were part of the core microbiome of cecal samples of house 2, but not of house 1. In litter samples, only 6 and 9 ASVs were part of the core microbiome in house 1 and 2, respectively (Supplementary Figure S4). The core ASVs found in the ceca of chickens from house 1 were associated with 15 families while the core ASVs from chickens in house 2 were classified into 7 families (Supplementary Table S4). The core ASVs in the litter of chickens from house 1 were classified into 3 families while ASVs in the litter from house 2 were grouped into 8 families.

We also determined the abundance of 16S rRNA gene reads that were associated with *Salmonella* or *Campylobacter* to see if there are differences between houses in the occurrence and abundance of food-borne pathogens. *Salmonella* was detected in the meconium and litter but not in the ceca and no reads were found for *Campylobacter*. For house 1, *Salmonella* was detected only in litter samples from day 14, while in house 2, it was found in litter samples from day 7, 14, 21, and 35 (Supplementary Table S5).

#### Temporal microbiome changes in the ceca and litter

Temporal shifts for individual microbiota members were separately determined for cecal and litter samples. In total, the relative abundance of 37 ASVs changed significantly over time in cecal samples (Supplementary Figure S5). Some genera comprised ASVs with diverging abundances. For example, *Alistipes* ASV 2168 was reduced over time, while others (ASVs 2,170, 2,173, 2,181) were significantly higher at later time points. Similarly, some ASVs of the genus *Bacteroides* increased while another ASV of this genus decreased. The *Escherichia/Shigella* ASV 1540 which was highly abundant in meconium samples decreased significantly over time in both cecal and litter samples but persisted until the end of the grow-out. Similarly, *Enterococcus* ASV 1097 was highly abundant in meconium samples, decreased but persisted in litter samples, but not in cecal samples. Overall, 5 ASVs decreased and 33 ASVs increased significantly throughout the course of the grow-out in litter samples (Supplementary Figure S6). Interestingly, ASV 1555, classified as *Klebsiella*, was the only ASV that showed an initial increase (from day 7 to day 14), before a subsequent decrease in abundance (day 7 compared to days 21, 28, 35, 42). Other ASVs that showed a reduction in abundance were associated with *Streptococcus*, *Lactobacillus*, and *Enterococcus*, while ASVs from the genera *Staphylococcus, Jeotgalicoccus, Facklamia, Brevibacterium, Corynebacterium, Brachybacterium,* and *Aerococcus* increased over time.

On day 49 feed was withdrawn from half of the chickens (*n* = 24) for 8 h to determine whether feed withdrawal affected the cecal microbiome of broiler chickens. Our analysis revealed that there was no significant difference (*p* > 0.05) in the cecal bacterial community structure between the feed and feed withdrawal group (Supplementary Figure S7).

#### AMR phenotype of *E. coli* and *Enterococcus* isolates

Although microbiome analysis informs us on the composition and relative abundance of bacterial species in a sample it lacks the resolution needed to infer strain level phenotypic differences. To get some insight on the phenotypic differences between bacterial strains from this study, we performed antimicrobial susceptibility testing on *E. coli* (*n* = 121) and *Enterococcus* strains (*n* = 115) recovered at the beginning (meconium samples) and end of the grow-out (day 49 cecal samples). We focused on antimicrobial resistance (AMR) because it is strongly correlated with the horizontal acquisition of antibiotic resistance genes and mutational resistance (Martinez and Baquero, 2000; Von Wintersdorff et al., 2016; Bortolaia et al., 2020). We used *E. coli and Enterococcus* because they had the highest relative abundance in the meconium of one day old chicks.

**FIGURE 7:**
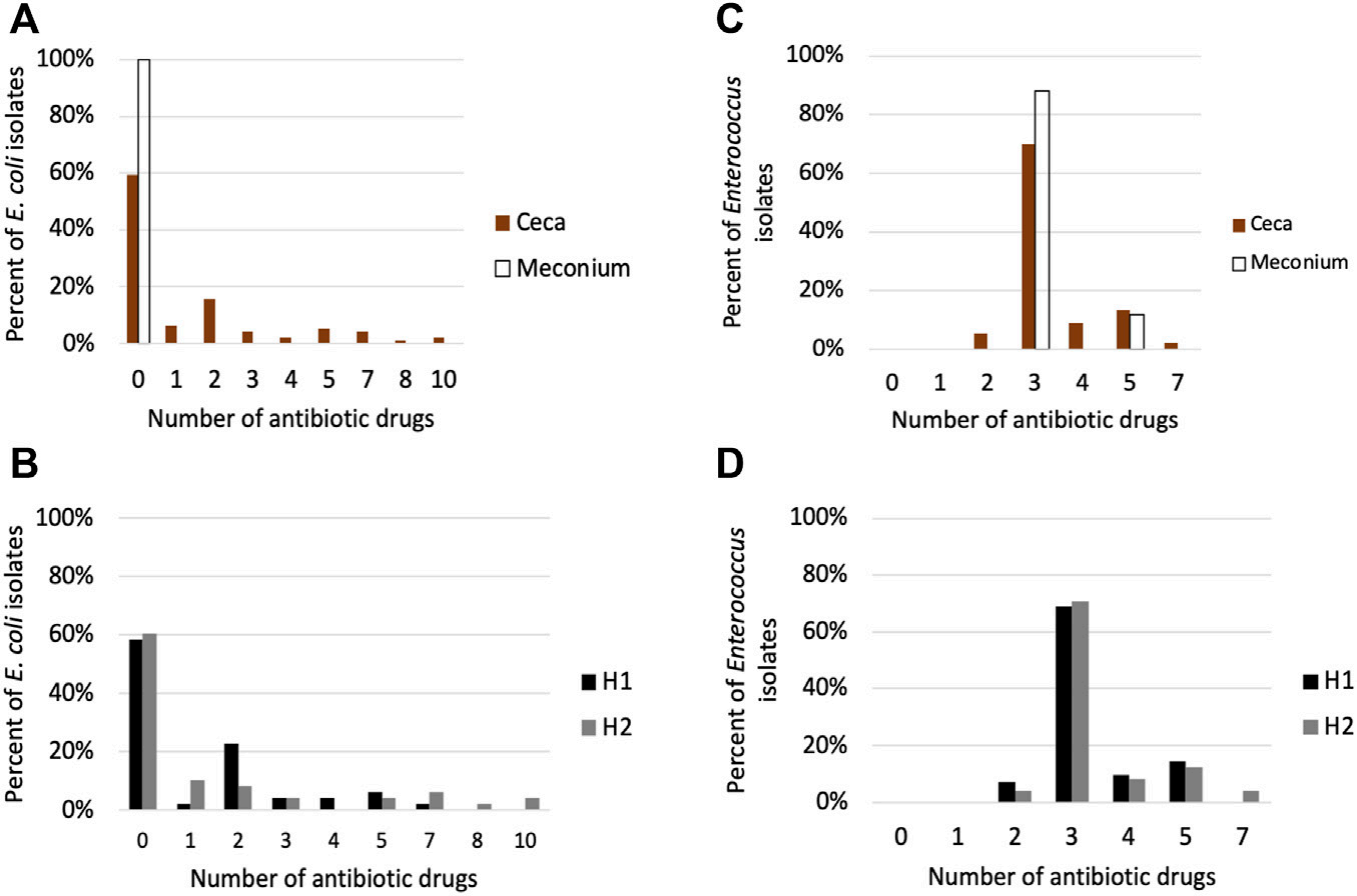
Antimicrobial susceptibility testing of (E) *coli* and *Enterococcu*s isolates. **(A)** Percentage of *E. coli* isolates in the meconium (*n* = 25) and ceca (*n* = 96) that are resistant to 0–10 antibiotics. **(B)** Percentage of cecal *E. coli* isolates from house 1 (*n* = 48) and house 2 (*n* = 48) that are resistant to 0–10 antibiotic drugs **(C)** Percentage of *Enterococcus* isolates in the meconium (*n* = 25) and ceca (*n* = 90) that are resistant to 0–10 antibiotics **(D)** Percentage of cecal *Enterococcus* isolates from house 1 (*n* = 42) and house 2 (*n* = 48) that are resistant to 0–10 antibiotic drugs.

All isolates were confirmed to be either *E. coli* or *Enterococcus* spp. using qPCR (Supplementary Table S1). The AMR phenotype of cecal *E. coli* isolates differed from the meconium isolates (*p* < 0.001). Meconium *E. coli* isolates were susceptible to all antibiotic drugs tested, while 40.6% of cecal isolates were resistant to 1–10 antibiotics belonging to 1–7 drug classes (Figure 7A). The most common resistance found were to tetracycline (*n* = 32), ampicillin, (*n* = 21), streptomycin (*n* = 22) and nalidixic acid (*n* = 15) (Supplementary Figure S8). There was no significant difference between the houses in the number of drug classes or drugs *E. coli* isolates were resistant to (*p* > 0.05), however, 6 of the 7 isolates that were resistant to 7–10 antibiotics were from cecal samples from house 2 (Figure 7B; Supplementary Figure S8).

Eighty-nine percent of *Enterococcus* isolates were *E. faecalis*, while *E. hirae* represented 2% of the isolates. We could not determine the species of *Enterococcus* for 11 isolates. There was no significant difference in AMR phenotype between meconium and cecal *Enterococcus* isolates (*p* > 0.05) (Figure 7C). *E. faecalis* isolates (101 of 102) were resistant to lincomycin, Synercid and tetracycline. Seventeen *E. faecalis* isolates displayed additional resistance to tylosin and erythromycin (Supplementary Figure S9). The two *E. hirae* isolates were resistant to lincomycin and tetracycline. Cecal *Enterococcus* isolates from house 1 differed from house 2 in the number of antibiotic drug classes they were resistant to (*p* < 0.05) (Figure 7D).

## Discussion

Early studies on the microbiome of the gastrointestinal tract of broiler chickens revealed that the core bacterial phyla in the GIT of broilers includes Firmicutes, Bacteriodota, Proteobacteria and Actinobacteriota (Oakley et al., 2014b; Borda-Molina et al., 2018; Feye et al., 2020) and that as chickens grow a successional change in bacterial composition and abundance is expected (Oakley et al., 2014a; Jurburg et al., 2019; Zhou Q. et al., 2021). Likewise, spatial differences in microbiome composition have been replicated across studies (Oakley et al., 2014b; Oakley and Kogut, 2016; Zhou Q. et al., 2021; Weinroth et al., 2022), and the microbiome diversity of the GIT and litter have been shown to be different (Cressman et al., 2010; Danzeisen et al., 2015; Wang et al., 2016). In this study, we present results that support some of these findings and provide new data on the environmental factors that are correlated with a change in the microbiome of broiler chickens. We found key differences in the relative abundance of the four core phyla between sample types. In the meconium Proteobacteria was the phylum with highest abundance, while Firmicutes dominated the ceca and litter microbiome. This difference in phyla abundance is expected since the environmental selective pressures present in each ecosystem are dissimilar.

Fertilized eggs to be hatched are incubated under warm temperatures (99–102°F) and are kept in clean/disinfected environments (Berrang et al., 2000; French, 2009; Archer and Cartwright, 2012; Wales and Davies, 2020). Therefore, the hatchery environment may select for bacterial species that can survive elevated temperatures and exposure to disinfectants. These surviving bacterial population would be the first colonizers of the GIT broiler chicks. Here, we found that members of the genera *Escherichia/Shigella* and *Enterococcus* were the main bacterial species in the meconium (the dark yellowish-green first droppings of a chick), of one-day old chicks. Jurburg et al. (2019) also reported that *Escherichia/Shigella* and *Streptococcus* were the major taxa found in fecal samples of one-day old broiler chicks. Similarly; Cárdenas-Rey et al. (2022), showed that *Escherichia/Shigella* was in high abundance in the ceca of one-day old broiler chicks (relative abundance of 37.3% ± 24.0%). Together, these results suggest that the bacterial taxa found in the meconium of day-one old chicks in the study were under selection in the hatchery.

After chicks were placed on pen floors in the broiler house, they were trained on how to drink from nipple waterers, and they began pecking at litter and feed. Therefore, it is plausible that they ingested microbes attached to physical, biological, and environmental matrices in the broiler house. Upon entry into the GIT, bacterial populations ingested are challenged with several selection pressures in the upper and lower GIT, including acidic pH, low oxygen levels, competition from resident microbiota and the chicken host immune responses. The cecum is part of the lower GIT that carries the highest bacterial densities, has the longest residence time of digesta, and is an important site for urea recycling and water regulation (Clench and Mathias, 1995; Oladeinde et al., 2019). In addition, bacterial populations in the ceca experience low redox potential that can lead to an increase in the abundance of obligate anaerobic bacteria and a reduction in aerobes or facultative anaerobes (Rinttilä and Apajalahti, 2013).

In this study, we found that members of the phylum Bacteriodota increased in abundance in the ceca (Figure 5). For instance, ASVs classified as *Barnesiella* and *Phascolarctobacterium* increased in the ceca from <1% relative abundance on day 14 to > 3.5% on days 42 and 49 (Supplementary Figures S5, S6). Members of these two bacterial taxa are obligate anaerobes that play a crucial role in the breakdown of carbohydrates and the production of short chain fatty acids (Weiss et al., 2014; Ikeyama et al., 2020). Contrastingly, we saw a significant decrease in the abundance of ASVs of facultative anaerobes such as *Escherichia/Shigella* and *Enterococcus* that were the dominant taxa in the meconium (Supplementary Figures S5, S6). Although, oxygen levels may have influenced the observed temporal changes in the abundance of the different taxa in ceca, other factors such as changes in diet at different chicken ages (i.e., starter diet from age 0–15, grower diet from age 15–29, and finisher diet from age 29–49) have been reported to affect microbial successional changes in the GIT of broiler chickens (Pan and Yu, 2014; Schokker et al., 2021). In fact, we found the age of chickens to be a significant factor that affected the bacterial beta-diversity in the ceca (Figure 3; Table 1).

The litter is a complex environment that is composed of decaying plant-based bedding material, feces, urine, feathers, and other broiler-sourced material. Furthermore, litter is exposed to broiler house environmental conditions such as temperature and relative humidity that are known to affect the physico-chemical characteristics of litter including litter moisture/water activity, pH, and ammonia. These environmental factors have been shown to affect the microbial community in litter (Ritz et al., 2005). Also, the bacterial population in litter are challenged with higher oxygen levels compared to the cecal microbiome. Therefore, it is not surprising that the relative abundance of strict anaerobes such as *Barnesiella* and *Phascolarctobacterium* decreased in litter, while aerobes (*Brachybacterium, Brevibacterium Corynebacterium* and *Luteimonas*) and facultative anaerobes (*Aerococcus, Facklamia, and Staphylococcus)* increased. These bacterial taxa displaced *Enterococcus* and *Escherichia/Shigella* in litter starting from day 14 and 21, respectively.

The majority of the ASVs that increased in the litter belonged to phylum Actinobacteria (Figure 5). Actinobacteria are known for their capability to biodegrade complex biopolymers and produce antimicrobials and bioactive substances (Vaijayanthi et al., 2016; Binda et al., 2018; van der Heul et al., 2018). For example, *Corynebacterium urealyticum* produces urease that catalyzes the hydrolysis of urea into carbon dioxide and ammonia (Salem et al., 2015; Gutierrez and Schneider, 2022), while some *Brevibacterium* spp. can efficiently degrade ammonia (Kim et al., 2013; Forquin and Weimer, 2014). In the current study, the relative abundance of *Corynebacterium* peaked between 21–28 days and coincided with the period that the highest broiler house ammonia levels were recorded (Supplementary Figure S3). Like the ceca, we also found that the day/age of broilers significantly affected the bacterial beta-diversity in the litter.

Broiler house environmental parameters (temperature, relative humidity, and ammonia) differed significantly between houses from day 22–day 49 of grow-out. House 2 had higher temperatures and higher ammonia in the mornings, while house 1 had higher relative humidity. Notably, these environmental changes coincided with a higher mortality and lower body weight of chickens in house 2. Likewise, we saw differences in the alpha and beta diversity of the ceca and litter microbiome between houses around this period (Table 2). For example, the alpha diversity of cecal samples decreased from day 28 to day 42 in house 2 but not in house 1, which suggests a perturbed gut microbiome (Pickard et al., 2017) (Supplementary Figure S1). Furthermore, we found core ASVs specific to the ceca and litter of each house (Supplementary Table S4). Elevated temperatures, atmospheric ammonia and relative humidity have been shown to increase mortality and reduce feed efficiency, body weight and feed intake in broilers (Weaver and Meijerhof, 1991; Miles et al., 2004; Vale et al., 2010; Wang et al., 2010; Alloui et al., 2011; Wasti et al., 2020). Heat stress and high ammonia have also been linked to a change in the microbiome of chickens (Shi et al., 2019; Zhou et al., 2021b; Liu et al., 2022). Taken together, these results suggest that there is a link between environmental conditions, an imbalance of the gut microbiome and poor broiler performance.

It is not clear why this difference in environmental conditions appeared after day 21 but we observed that the outside temperature from day 1–21 (average highs: 93.22 ± 3.54°F; range: 86–99°F) was higher than day 22–49 (average highs: 91.3 ± 4.74°F; range: 79–97°F) (https://www.ncdc.noaa.gov/cdo-web/; weather station: USW00013873, Athens Ben Epps Airport, Georgia, United States, 33.94773,-83.32736). The climate and weather outside can significantly influence broiler house environmental parameters and proper ventilation is one of the best management practices recommended to ensure that house conditions are optimal for broiler welfare and health. In this study, we used Portacool fans during hot weather days to reduce the temperature inside the house. Therefore, one or both variables (i.e., hot weather and use of Portacool fans) may have contributed to the differences in environmental conditions between houses. It is not clear whether the differences in environmental conditions caused changes in the microbiome, or if they just coincided. Non-etheless, we did observe not only changes in relative abundances, but also differences in the core microbiome between the two houses.

For instance, the ceca of chickens from house 2 harbored four core ASVs classified as Order_Clostridia_vadinBB60_group (Supplementary Table S4). This group of bacteria are not well classified, and little is known about their metabolism or role in the microbiota (Richards et al., 2019). Zhou et al. (2017) reported an increase in the abundance of order_Clostridia_vadinBB60_group in the ceca of chickens infected with *Eimeria tenella*. Also, we have shown that members of the Order_Clostridia_vadinBB60_group increased in abundance in the ceca and litter of broiler chicks infected with *Salmonella* Heidelberg and raised on fresh pine shavings (Oladeinde et al., 2022). Furthermore, three core ASVs found in the litter of chickens from house 1 but not in the litter from house 2 were classified as *Lactobacillus* including *L. johnsonii* (Supplementary Table S4). *Lactobacillus* spp. are regarded as safe and beneficial microbes and have been extensively employed in the development of probiotics (Zhang et al., 2018). Additionally, we found *Salmonella* 16S rRNA gene reads in the litter from house 2 during four sampling time points while *Salmonella* was detected only in day 14 litter samples from house 1 (Supplementary Table S5). The lack of *Salmonella* 16S rRNA gene reads in cecal samples was unexpected and suggest that *Salmonella* was in low abundance and/or in a non-viable state in the litter. Bacterial cell viability and inoculum concentration could affect the rate *Salmonella* colonizes the GIT of chickens. Taken together, these results suggest that the environment and the microbiome in house 1 was different from house 2.

Lastly, we found that the prevalence of AMR in *E. coli* isolates differed between meconium and ceca. Horizontal gene transfer is the main mechanism bacteria acquires AMR genes (Von Wintersdorff et al., 2016). *E. coli* isolates recovered from the meconium were susceptible to all antibiotics tested, while ∼41% of cecal *E. coli* isolates were resistant to at least 1 antibiotic (Figure 7A). Contrastingly, there was no significant difference in AMR prevalence between meconium and cecal *Enterococcus* isolates suggesting that limited HGT of AMR occurred in *Enterococcus* isolates. All *E. faecalis* isolates from the meconium (*n* = 23) and 99% of cecal isolates (*n* = 78) were resistant to lincomycin, Synercid (quinupristin/dalfopristin) and tetracycline and only three cecal *E. faecalis* isolates displayed resistance to additional antibiotics that were not seen in meconium isolates. It is possible that *E. coli* and *Enterococcus* isolates selected from the meconium are not representative of all AMR phenotypes present in one-day old broilers.

In conclusion, this study showed that the microbiome of the ceca and litter of broiler chickens changed over time. Furthermore, differences in microbiome between houses were correlated with changes in house environmental parameters. However, since our study has no repeatability and environmental conditions were not controlled, additional studies are necessary to investigate whether this is generally true, or it is specific only to the broiler houses in this study. Therefore, it is crucial that animal studies pay close attention to environmental differences between houses/barns/cages as this can potentially be a source of confounders and introduce variability in experimental outcomes.

## Data Availability

The datasets presented in this study can be found in online repositories. 16S rRNA gene sequences are publicly available under NCBI accession no: PRJNA699167.

## Supplementary material

Supplementary Figure S1 Change of alpha diversity indices from rarefied 16S rRNA gene libraries between houses. Dots represent values from individual days, lines represent loess curves of the moving average, and shaded areas indicate the confidence interval (0.95).

Supplementary Figure S2 Chicken body weight **(A)** and cumulative mortality **(B)** throughout the course of the study.

Supplementary Figure S3 Measurement of broiler house and litter environmental parameters. Boxes indicate the interquartile range (from 75 to 25th) of the data. Whiskers extend to the most extreme value within 1.5 times interquartile range and dots represent outliers beyond that range. Only significant changes are shown with asterisks: *: p ≤ 0.05, **:p ≤ 0.01. House temperature and relative humidity were measured daily, while NH3, pH and moisture were measured weekly from pooled litter from six pens per house.

Supplementary Figure S4 Venn diagram showing the number of core and non-core ASVs found in the ceca and litter of broilers.

Supplementary Figure 5 Heatmap illustrating temporal microbial shifts in cecal samples. Changes in microbial abundance were computed between day 7 and each of the other days separately. The effect size depicts the negative log of the q-value multiplied by the sign of the coefficient. Taxonomy of the ASVs is indicated at the genus levels or at the lowest rank that could be assigned confidently (Bootstrap support above 50). Only significant changes are shown.

Supplementary Figure S6 Heatmap illustrating temporal microbial shifts in litter samples. Changes in microbial abundance were computed between day 7 and each of the other days separately. The effect size depicts the negative log of the q-value multiplied by the sign of the coefficient. Taxonomy of the ASVs is indicated at the genus levels or at the lowest rank that could be assigned confidently (Bootstrap support above 50). Only significant changes are shown.

Supplementary Figure S7 Principal component analysis of bacterial communities from cecal samples on day 49. Points indicate samples from chickens raised in house H1 and triangles show samples from chickens raised in house H2. Samples from chickens with or without feed withdrawal are illustrated in blue and red respectively.

Supplementary Figure S8 Heatmap of the antimicrobial resistance phenotype of E. coli isolates. Each row represents one isolate tested for susceptibility against the CMV4AGNF panel. Columns on left hand side represent the isolate associated metadata. Number of antibiotics reflects the total number of antibiotics an isolate was resistant to on the panel and number of drug classes refers to the enumeration of antibiotics an isolate is resistant to on the panel of the same drug class. AMP, Ampicillin; TET, Tetracycline; GEN, Gentamicin; SOX, Sulfizoxazole; FOX, Cefoxitin; AMC, Amoxicillin–Clavulanic Acid; AXO, Ceftriaxone; SXT, Trimethoprim– Sulfamethoxazole; AZM, Azithromycin; CHL, Chloramphenicol; NAL, Nalidixic acid; STR, Streptomycin.

Supplementary Figure S9 Heatmap of the antimicrobial resistance phenotype of Enterococcus Isolates. Each row represents one isolate tested for susceptibility against the CMV3AGPF panel. Columns on left hand side represent the isolate associated metadata. Number of antibiotics reflects the total number of antibiotics an isolate was resistant to on the panel and number of drug classes refers to the enumeration of antibiotics an isolate is resistant to on the panel of the same drug class. LIN, Lincomycin; SYN, Synercid (Quinupristin/ Dalfopristin); TET, Tetracycline; ERY, Erythromycin; TYL, Tylosin; GEN, Gentamicin; KAN, Kanamycin; CIP, Ciprofloxacin; NIT, Nitrofurantoin.
