# Supplementary material for: Temporal dynamics of the cecal and litter microbiome of chickens raised in two separate broiler houses
Source: Front Physiol. 2023 Mar 2;14:1083192. doi: 10.3389/fphys.2023.1083192 (PMC10018173; doi:10.3389/fphys.2023.1083192)
Supplement: Supplementary file 2 [file Table1.DOCX]

Supplementary Table 1: Primers used for *E. coli* and *Enterococcus* species confirmation and identification.

| Assay | Primer | Sequence | Target | Reference |
| --- | --- | --- | --- | --- |
| EPA-EC 23S | Forward | GGTAGAGCACTGTTTTGGCA | *E. coli* | Chern et al., 2011 |
|  | Reverse | TGTCTCCCGTGATAACTTTCTC |  |  |
|  |  |  |  |  |
| Entero1 | Forward | AGAAATTCCAAACGAACTTG | *Enterococcus* spp. | Ludwig et al., 2000 |
|  | Reverse | CAGTGCTCTACCTCCATCATT |  |  |
|  |  |  |  |  |
| *E. faecalis* | Forward | ACTTATGTGACTAACTTAACC | *E. faecalis* | Jackson et al., 2004 |
|  | Reverse | TAATGGTGAATCTTGGTTTGG |  |  |
|  |  |  |  |  |
| *E. faecium* | Forward | GAAAAAACAATAGAAGAATTAT | *E. faecium* | Jackson et al., 2004 |
|  | Reverse | TGCTTTTTTGAATTCTTCTTTA |  |  |
|  |  |  |  |  |
| *E. hirae* | Forward | CTTTCTGATATGGATGCTGTC | *E. hirae* | Jackson et al., 2004 |
|  | Reverse | TAAATTCTTCCTTAAATGTTG |  |  |
