# Supplementary figures and images for: Temporal dynamics of the cecal and litter microbiome of chickens raised in two separate broiler houses
Source: Front Physiol. 2023 Mar 2;14:1083192. doi: 10.3389/fphys.2023.1083192 (PMC10018173; doi:10.3389/fphys.2023.1083192)

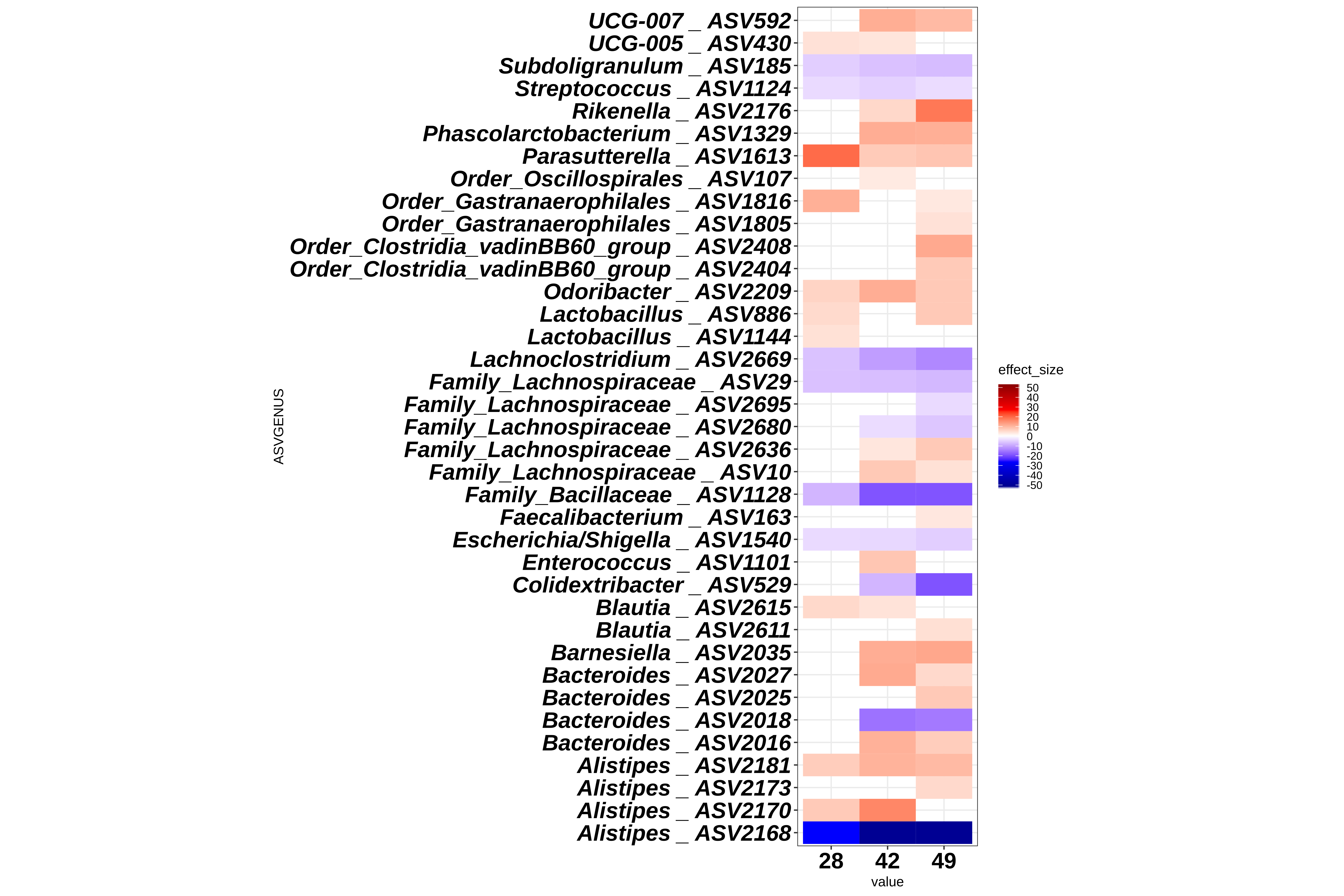

Supplement: Supplementary file 4 [file Image5.TIFF]

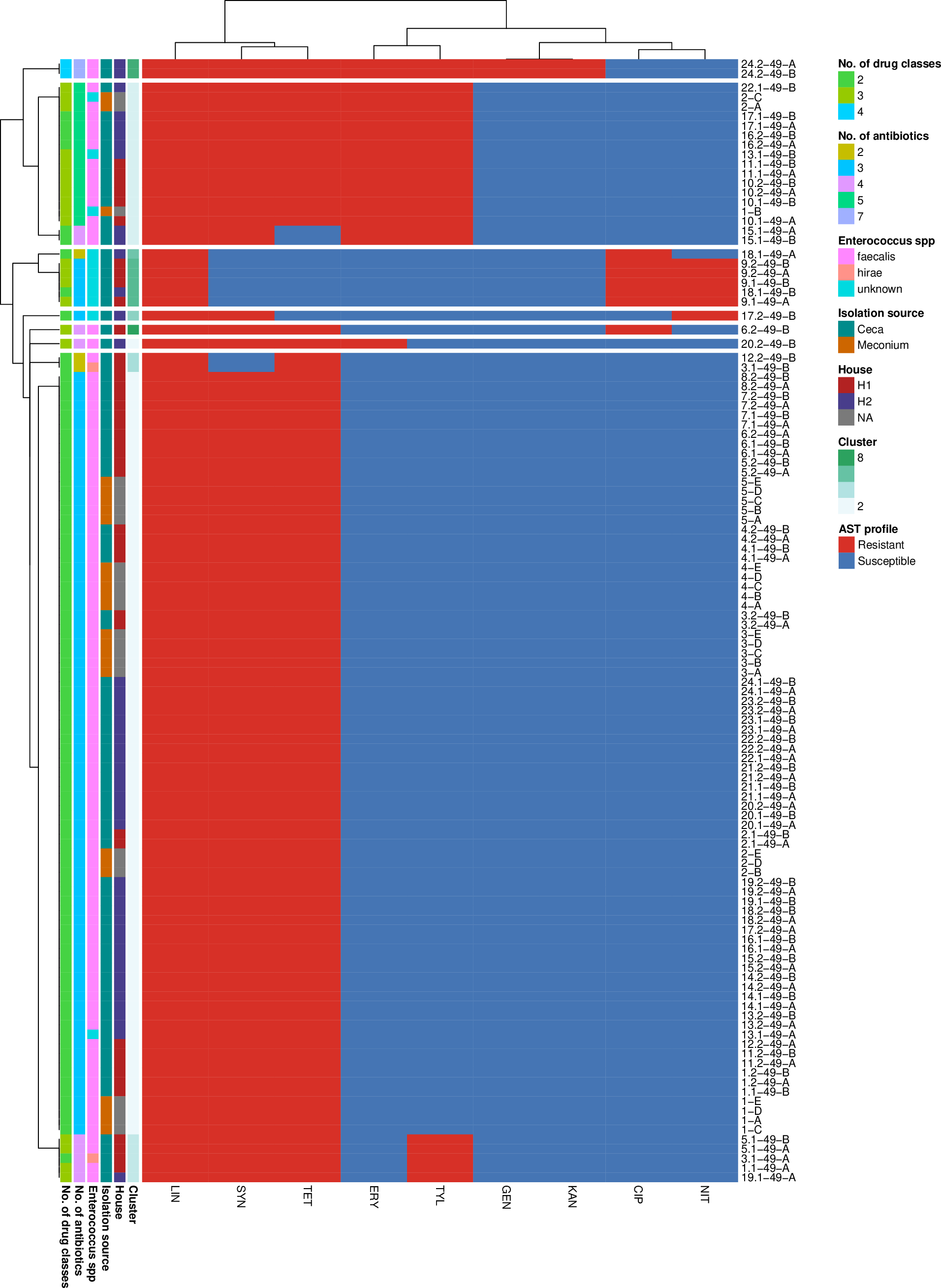

Supplement: Supplementary file 5 [file Image9.TIF]

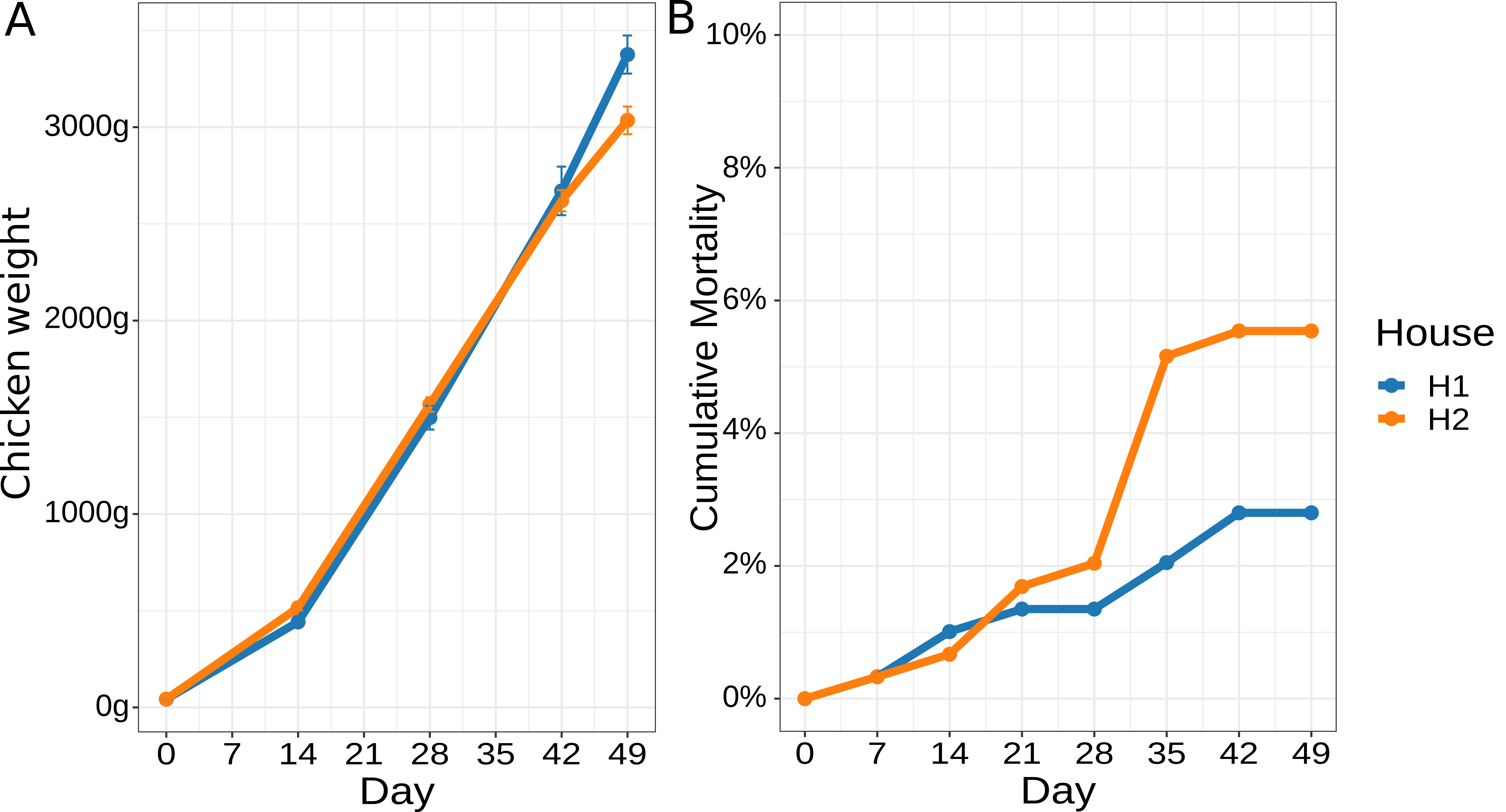

Supplement: Supplementary file 6 [file Image2.TIF]

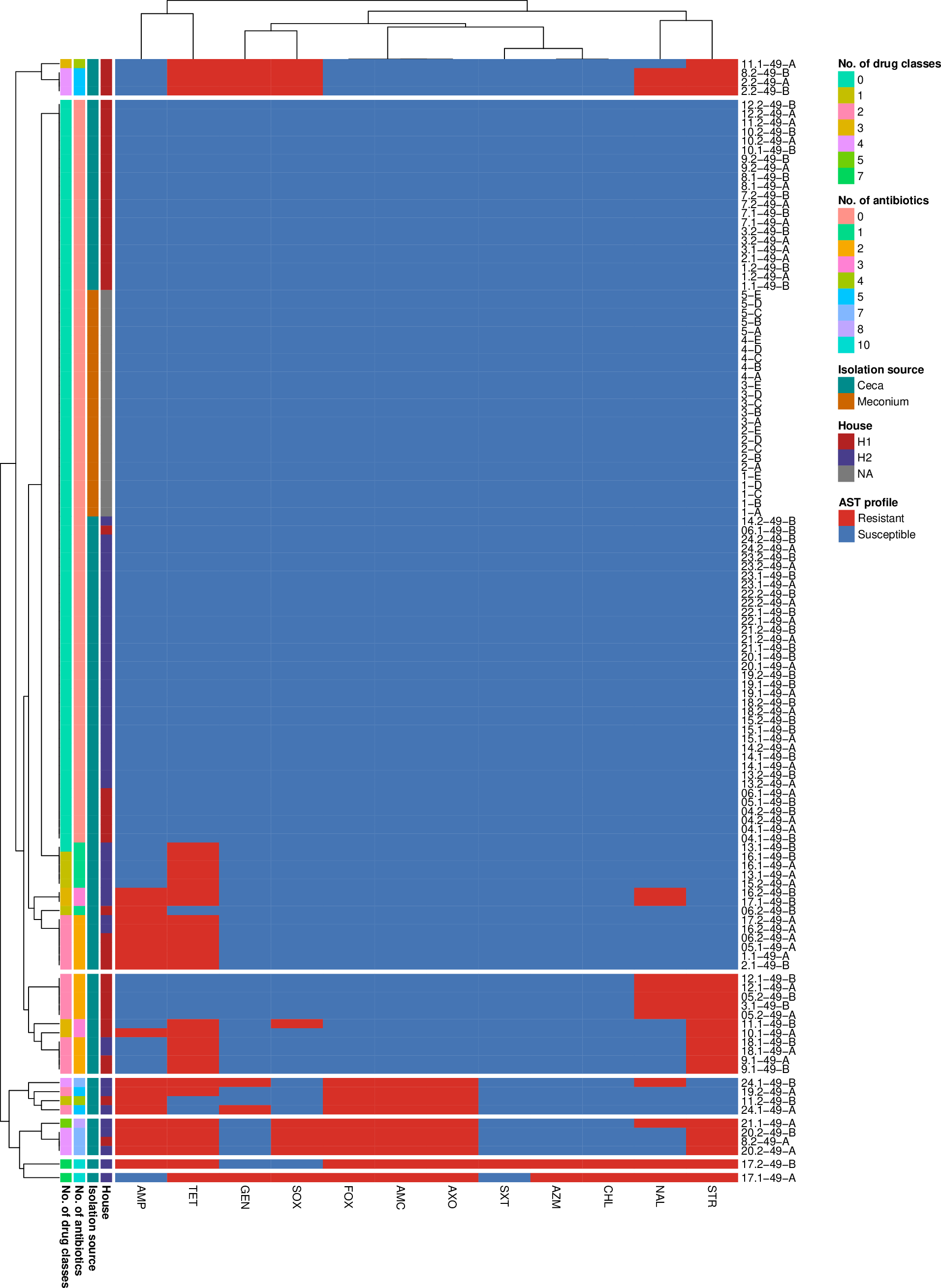

Supplement: Supplementary file 11 [file Image8.TIF]

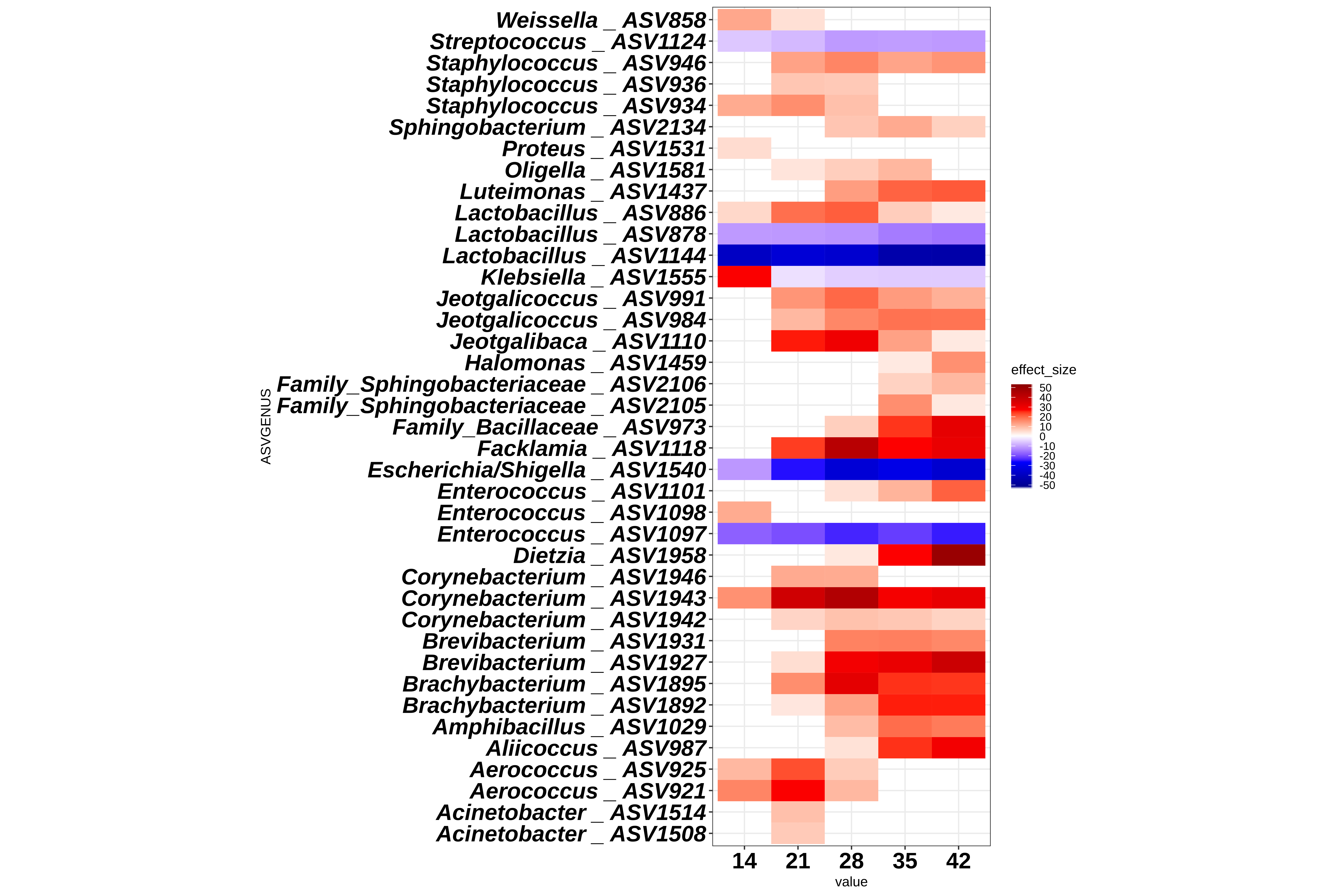

Supplement: Supplementary file 12 [file Image6.TIFF]

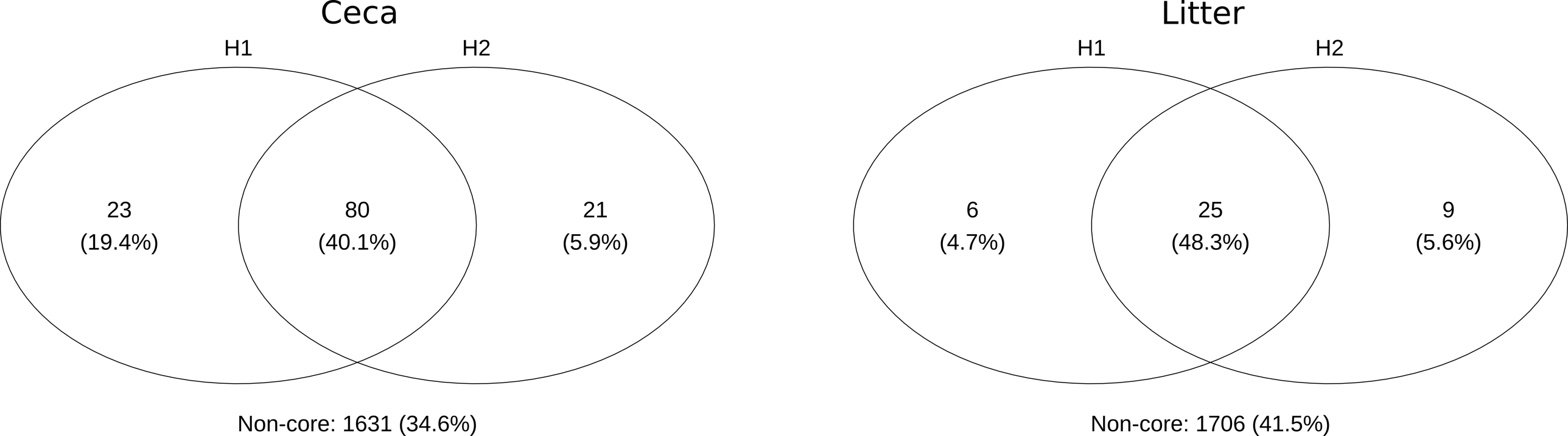

Supplement: Supplementary file 13 [file Image4.TIFF]

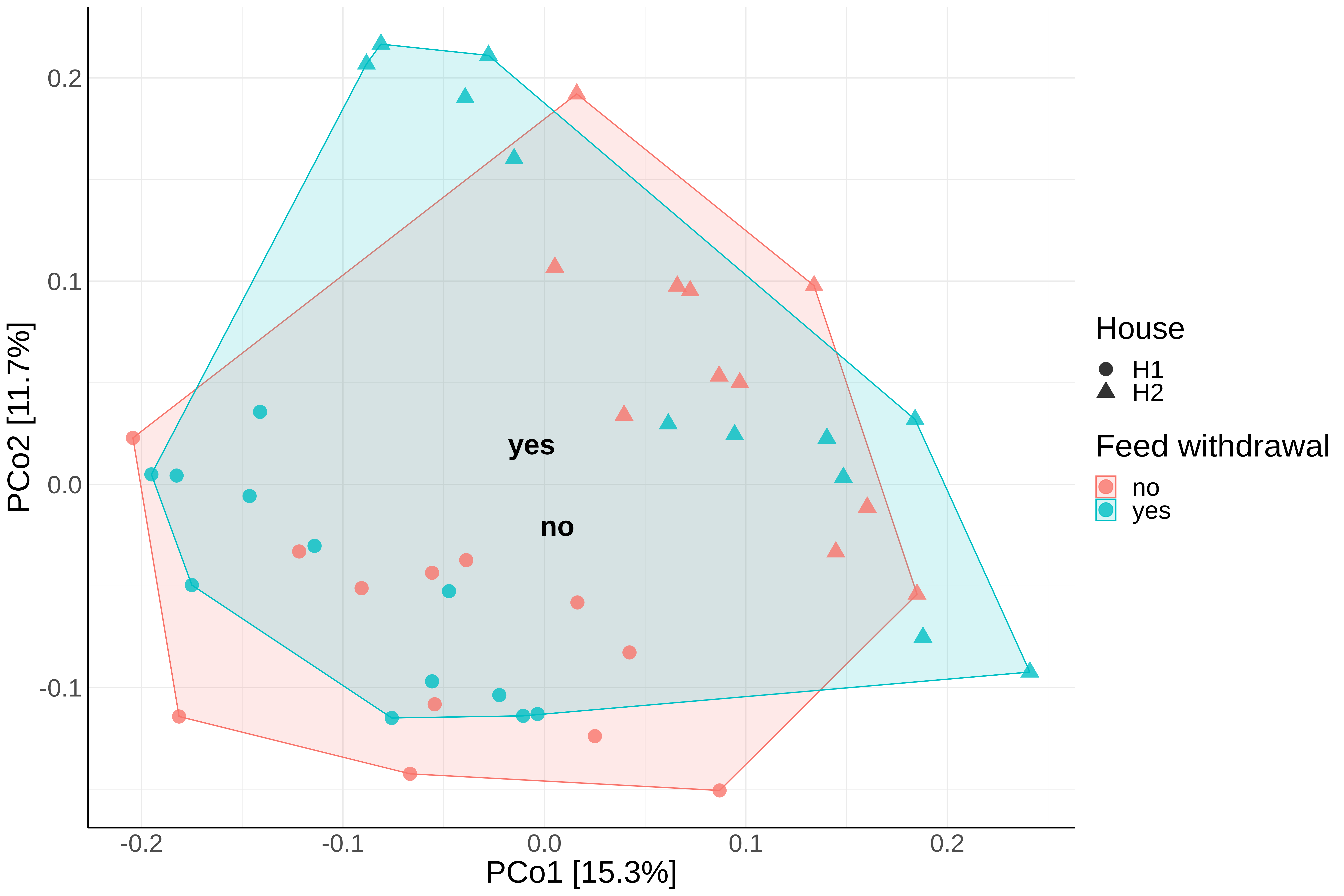

Supplement: Supplementary file 14 [file Image7.TIFF]
