# Supplementary material for: Temporal dynamics of the cecal and litter microbiome of chickens raised in two separate broiler houses
Source: Front Physiol. 2023 Mar 2;14:1083192. doi: 10.3389/fphys.2023.1083192 (PMC10018173; doi:10.3389/fphys.2023.1083192)
Supplement: Supplementary file 8 [file Table2.DOCX]

**Supplementary Table 2** : Statistical comparison of environmental parameters between houses.

| Day | Group1^a^ | Group2^a^ | P. value | Significance^b^ | Method | Variable |
| --- | --- | --- | --- | --- | --- | --- |
| 0-21 | H1 | H2 | 0.7519929123 | ns | Wilcoxon | Temperature AM |
| 22-49 | H1 | H2 | 0.002871360168 | ** | Wilcoxon | Temperature AM |
| 0-21 | H1 | H2 | 0.6629928927 | ns | T-test | Temperature PM |
| 22-49 | H1 | H2 | 0.8572680956 | ns | T-test | Temperature PM |
| 0-21 | H1 | H2 | 0.8304000051 | ns | Wilcoxon | Humidity AM |
| 22-49 | H1 | H2 | 0.02840045285 | * | Wilcoxon | Humidity AM |
| 0-21 | H1 | H2 | 0.6257765558 | ns | Wilcoxon | Humidity PM |
| 22-49 | H1 | H2 | 0.947652163 | ns | Wilcoxon | Humidity PM |
| 0-21 | H1 | H2 | 0.553616992 | ns | Wilcoxon | Ammonia ppm/h |
| 22-49 | H1 | H2 | 0.04112554113 | * | Wilcoxon | Ammonia ppm/h |
| 0-21 | H1 | H2 | 0.6666666667 | ns | Wilcoxon | pH |
| 22-49 | H1 | H2 | 0.6004018481 | ns | Wilcoxon | pH |
| 0-21 | H1 | H2 | 1 | ns | Wilcoxon | Moisture per room |
| 22-49 | H1 | H2 | 1 | ns | Wilcoxon | Moisture per room |

^a^H1, House 1; H2, House 2

^b^ ns, not significant, *, P ≤ 0.05; **; P ≤ 0.01
