# Supplementary material for: Temporal dynamics of the cecal and litter microbiome of chickens raised in two separate broiler houses
Source: Front Physiol. 2023 Mar 2;14:1083192. doi: 10.3389/fphys.2023.1083192 (PMC10018173; doi:10.3389/fphys.2023.1083192)
Supplement: Supplementary file 9 [file Table5.DOCX]

Supplementary Table 5: *Salmonella* 16S rRNA gene read abundance in cecal and litter samples

| Day | Sample type | House^a^ | Average | Standard deviation |
| --- | --- | --- | --- | --- |
| 0 | Meconium | NA | 3 | 1.414213562 |
| 14 | ceca | H1 | 0 | 0 |
| 28 | ceca | H1 | 0 | 0 |
| 42 | ceca | H1 | 0 | 0 |
| 49 | ceca | H1 | 0 | 0 |
| 14 | ceca | H2 | 0 | 0 |
| 28 | ceca | H2 | 0 | 0 |
| 42 | ceca | H2 | 0 | 0 |
| 49 | ceca | H2 | 0 | 0 |
| 7 | litter | H1 | 0 | 0 |
| 14 | litter | H1 | 33.3333333 | 81.64965809 |
| 21 | litter | H1 | 0 | 0 |
| 28 | litter | H1 | 0 | 0 |
| 35 | litter | H1 | 0 | 0 |
| 42 | litter | H1 | 0 | 0 |
| 7 | litter | H2 | 5.83333333 | 9.8877028 |
| 14 | litter | H2 | 55 | 82.10237512 |
| 21 | litter | H2 | 3 | 4.69041576 |
| 28 | litter | H2 | 0 | 0 |
| 35 | litter | H2 | 5.83333333 | 12.87504045 |
| 42 | litter | H2 | 0 | 0 |

^a^NA, not applicable; H1, House 1; H2, House 2
